# Agreement between clinicians' and care givers' assessment of intelligence in Nigerian children with intellectual disability: 'ratio IQ' as a viable option in the absence of standardized 'deviance IQ' tests in sub-Saharan Africa

**DOI:** 10.1186/1744-9081-5-39

**Published:** 2009-09-15

**Authors:** Muideen O Bakare, Vincent N Ubochi, Ifeoma N Okoroikpa, Chinyere M Aguocha, Peter O Ebigbo

**Affiliations:** 1 Child and Adolescent Unit, Federal Neuro-Psychiatric Hospital, New Haven, Enugu, Enugu State, Nigeria; 2 Therapeutic Care Centre (TCC), Abakpa, Nike, Enugu, Enugu State, Nigeria; 3 Department of Psychological Medicine, University of Nigeria Teaching Hospital, (UNTH), Enugu, Enugu State, Nigeria

## Abstract

**Background:**

There may be need to assess intelligent quotient (IQ) scores in sub-Saharan African children with intellectual disability, either for the purpose of educational needs assessment or research. However, modern intelligence scales developed in the western parts of the world suffer limitation of widespread use because of the influence of socio-cultural variations across the world. This study examined the agreement between IQ scores estimation among Nigerian children with intellectual disability using clinicians' judgment based on International Classification of Diseases, tenth Edition

(ICD - 10) criteria for mental retardation and caregivers judgment based on 'ratio IQ' scores calculated from estimated mental age in the context of socio-cultural milieu of the children. It proposed a viable option of IQ score assessment among sub-Saharan African children with intellectual disability, using a ratio of culture-specific estimated mental age and chronological age of the child in the absence of standardized alternatives, borne out of great diversity in socio-cultural context of sub-Saharan Africa.

**Methods:**

Clinicians and care-givers independently assessed the children in relation to their socio-cultural background. Clinicians assessed the IQ scores of the children based on the ICD - 10 diagnostic criteria for mental retardation. 'Ratio IQ' scores were calculated from the ratio of estimated mental age and chronological age of each child. The IQ scores as assessed by the clinicians were then compared with the 'ratio IQ' scores using correlation statistics.

**Results:**

A total of forty-four (44) children with intellectual disability were assessed. There was a significant correlation between clinicians' assessed IQ scores and the 'ratio IQ' scores employing zero order correlation without controlling for the chronological age of the children (r = 0.47, df = 42, p = 0.001). First order correlation controlling for the chronological age of the children showed higher correlation score between clinicians' assessed IQ scores and 'ratio IQ' scores (r = 0.75, df = 41, p = 0.000).

**Conclusion:**

Agreement between clinicians' assessed IQ scores and 'ratio IQ' scores was good. 'Ratio IQ' test would provide a viable option of assessing IQ scores in sub-Saharan African children with intellectual disability in the absence of culture-appropriate standardized intelligence scales, which is often the case because of great diversity in socio-cultural structures of sub-Saharan Africa.

## Background

### Advent and history of human intelligence testing

One of the earliest objectives of developing scales to assess intelligence among children was to determine those children that would require special attention and instructions in educational systems. Alfred Binet, the French psychologist published the first modern intelligence test called Binet-Simon intelligence scale in 1904 [1-3].

William Stern, the German Psychologist coined the abbreviation, 'IQ (intelligence quotient)' in 1912, with a proposition that individual's intelligence level can be measured as a quotient of mental age and chronological age. This proposition was adapted into the revised Binet-Simon scale which became the Stanford-Binet scale which was published in 1916 by Lewis Terman from Stanford University. The Stanford-Binet intelligence scale led to a mental age score and IQ was calculated as a percentage of mental age and chronological age ratio [2,3].

Subsequently over the years, standardized intelligence scales that based IQ scoring on normal distribution curve rather than age-based quotient had been developed [3]. The newer standardized intelligence scales widely used in the western parts of the world employed the use of Gaussian bell curve with an average IQ of 100 as the center value and standard deviation of 15 or occasionally 16 or 24 points on either sides of the Gaussian bell curve [3]. These modern intelligence scale scoring method that employed the use of standard deviation on bell curve are sometimes referred to as 'deviance IQ' as against the former scale which measured IQ in ratio of mental age and chronological age and sometimes referred to as 'ratio IQ'.

The modern standardized intelligence scales like Wechsler Intelligence Scales for Children (WISC - R, WISC - III and WISC - IV) had been known to be influenced by socio-cultural variations in different environments that impact on child rearing practices and cultural expectations of a child in a particular society [4-6].

### Nature, nurture and human intelligence

It is well established that both genes and environments have influence on cognitive abilities [7,8]. Factors influencing human intelligence had been noted to be multiple and include social, environmental, neural and genetic factors [7]. Therefore, analysis of human intelligence should be based on these multiple level of factors [7]. Controversy still exists on how much nurture as compared to nature contributes to apparent racial and cultural differences in IQ scores [9-13]. However, that culture, environment and ethnic background influence human intelligence remained an undisputable fact. Han and Northoff [14] in a recent trans-cultural neuro-imaging study demonstrated that cultural background can influence the neural activity that underlies cognitive functions. They proposed that the approach of trans-cultural neuro-imaging may provide means of distinguishing culture-sensitive from culture-invariant neural mechanisms of human cognition [14].

### Ethnic and cultural diversity in sub-Saharan Africa

The great diverse multi-ethnic and multi-culture nature of sub-Saharan African regions are often impediments to developing comparable standardized IQ assessment scales among children that would cut across sub-cultures as obtained in the Western region of the world. Nigeria for example, which is a country in sub-Saharan Africa has well over three hundred major and minor ethnic tribes and sub-cultures [15,16] that could impact largely on child rearing practices and socio-cultural expectations of the child in the society.

The attendant influence of socio-cultural variations across regions on cognitive ability and social competence of children, necessitated the provision of a general guide by Diagnostic Criteria for Research of International Classification of Diseases, tenth Edition (ICD - 10) [17] for assessing mental retardation among children with intellectual disability across cultures.

This study assessed the agreement between 'ratio IQ' scores calculated from ratio of mental age and chronological age of children with intellectual disability as assessed by nursing staff and teacher (care givers) and clinicians' assessed IQ scores, based on the categories specified in the Diagnostic Research Criteria of ICD - 10 for mental retardation [17].

## Methods

### Location and participants

The location of the study was the Therapeutic Care Center (TCC), Abakpa, Enugu, Nigeria. This privately owned center provides special educational instructions, and behavioral modification to children with intellectual disabilities in south eastern Nigeria. The participants are children with intellectual disability admitted into the boarding facility of the TCC, Abakpa, Enugu, Nigeria. All children with suspected learning disability admitted into the center were studied. The children have associated co-morbid medical conditions ranging from Pervasive developmental disorders (PDD), Down syndrome and Cerebral palsy.

### Ethical consideration

Ethical approval for this study was obtained from the Institutional Review Board (IRB) of Federal Neuro-Psychiatric Hospital, New Haven, Enugu, Enugu State, Nigeria. Informed consent was obtained from the parents of the children studied and from the authority of Therapeutic Care Center (TCC), Abakpa, Enugu, Nigeria.

## Materials

### Socio-demographic questionnaire

This questionnaire was designed to obtain socio-demographic information of the children, which included their chronological or actual age and mental age as estimated by the consensus agreement of the nursing staff and teacher (care givers) who are of the same socio-cultural background with the children and responsible for the care of each child studied at the TCC, Abakpa, Enugu, Nigeria. From the information obtained about the estimated mental age and chronological age of the children, 'ratio IQ' score was calculated for each child using a ratio of culture-specific estimated mental age and chronological age multiply by a factor of 100.

### ICD - 10 diagnostic criteria for mental retardation [17]

The diagnostic criteria stated that depending upon the cultural norms and expectations of the individuals being studied; research workers must make their own judgments as to how best to estimate intelligence quotient (IQ) or mental age according to the bands given in Table 1.

**Table 1:** ICD - 10 diagnostic criteria for mental retardation

**Category**	**Mental Retardation**	**IQ Range**	**Median IQ**	**Mental Age Range (Years)**	**Median Mental Age**
**F 70**	Mild	50 - 69	60	9 to under 12 (9 - 11)	10

**F 71**	Moderate	35 - 49	42	6 to under 9 (6 - 8)	7

**F 72**	Severe	20 - 34	27	3 to under 6 (3 - 5)	4

**F 73**	Profound	Below 20	10	Less than 3 (0 - 2)	1

Two clinicians that are of the same socio-cultural background with the children independently assessed each child with the diagnostic criteria in Table 1 and consensus was reached between them. Each child was allocated to specific categories in Table 1, either as profound mental retardation, severe mental retardation, moderate mental retardation or mild mental retardation based on the clinicians' judgment. Each child assigned a specific category in Table 1 was also given a median IQ score and median mental age in the particular category.

### Procedure

The socio-demographic questionnaire was used to obtain information from which 'ratio IQ' score was calculated for each child. Each child was also assigned IQ scores based on the clinicians' assessment and placement of the child in specific diagnostic categories as spelt out in ICD - 10 diagnostic criteria [17] shown in Table 1. The nursing staffs, teachers and clinicians involved in the assessment of the children had been providing routine services to the children at the TCC, Abakpa, Enugu, Nigeria for at least a period of six months. They were of the same socio-cultural background as the children and were also fluent in the language and dialect of the children. They belong to Igbo ethnic group in south eastern region of Nigeria. The nursing staffs possessed minimum qualification of diploma in nursing, while the teachers also possessed minimum of diploma qualification in special education and were familiar with background knowledge of normal child development.

### Criteria followed for assessment of mental age by the nursing staffs and teachers

Two major specific areas were assessed to estimate the mental age of the children which are:

**i. Cognitive ability **- This was assessed based on age appropriate;

• Ability of the child to communicate, being able to pronounce words, phrases and sentences that are comprehensible and appropriate for age - **Verbal test**.

• Ability of the child to copy and draw shaped objects such as circles, squares and triangles - **Performance test**.

**ii. Social competence **- This was assessed based on the age appropriate;

• Ability of the child to care for self, which includes cleaning up self and dressing for classes in the morning. This was rated in the range of, Need hundred percent help in caring for self; Need substantial help in caring for self and, Need minimal help in caring for self - **Adaptive self care**.

• Ability of the assessed children to relate with other pupils and teachers in the areas of expressing their needs and wants - **Adaptive social skills**.

### Criteria followed by the clinicians in assigning each child to specific ICD-10 diagnostic category of mental retardation

The criteria followed by the clinicians in assigning the assessed children to specific diagnostic category of ICD-10 criteria for mental retardation were essentially the same as that followed by the teachers and nursing staffs in estimating the mental age, with the exemption that assessment of social competence by the clinicians were based on observation of the children's ability to relate with other pupils and the teachers to express their needs and wants.

The clinicians involved in assessment of the children are psychiatrist trainees at Senior Registrar level of their training and had been working with children with learning disabilities for a minimum of six months period. All assessment by the clinicians, teachers and the nursing staffs were based on the same specific socio-cultural milieu with the children assessed.

### Difference between care givers' and clinicians' assessment of IQ in the children

The major difference between the care givers' and clinicians' assessment is that the clinicians had access to categories of ICD-10 diagnostic criteria for mental retardation and based their assessment on this, but the care givers did not. The IQ of each child was assessed by the care givers from a ratio of estimated mental age and the chronological age of the child.

### Data analysis

Data were analyzed using the Statistical Package for Social Sciences (SPSS), version 15.

The frequency and percentage distribution of the children in different diagnostic categories of mental retardation as specified by ICD - 10 [17] based on the clinicians assessment and calculated 'ratio IQ' scores were computed and difference in the distribution frequency among the categories was tested with Chi-square statistics. The correlations between mean 'ratio IQ' scores and mean IQ scores based on clinicians' judgment were also tested with and without controlling for the chronological age of the children using partial correlation statistics.

## Results

### Socio-demographic variables

A total of forty four (44) children with intellectual disability were independently assessed by the care-givers and the clinicians for estimation of mental age and classification under different categories of ICD - 10 diagnostic criteria for mental retardation [17]. There were 28 (63.6%) males and 16 (36.4%) females.

The actual or chronological age range of the children was from 4 to 18 years. The mean chronological age of the children was 13.16 ± 3.58 years. The mean mental age as assessed by the care-givers was 4.82 ± 1.90 years. The mean mental age as assessed by the clinicians using ICD - 10 [17] diagnostic criteria for mental retardation was 4.75 ± 2.06 years. The mean 'ratio IQ' scores as calculated from ratio of mental age and chronological age was 38.14 ± 12.81. The mean IQ scores as assessed by the clinicians based on ICD - 10 [17] diagnostic criteria for mental retardation using the median scores in each diagnostic category was 30.71 ± 11.00. The socio-demographic variables of the children are shown in Table 2.

**Table 2:** Socio-demographic variables of the children

**Socio-demographic Variables**	**Values**
**Gender {N (%)}**	
Male	28 (63.6)
Female	16 (36.4)

**Chronological/Actual Age (Years)**	
Range	4 - 18
Mean	13.16 ± 3.58

**Mental Age (Care Givers' Assessment) (Years)**	
Mean	4.82 ± 1.90

**Mental Age (Clinicians' Assessment) (Years)**	
Mean	4.75 ± 2.06

**Ratio IQ Score (Care Givers' Assessment)**	
Mean	38.14 ± 12.81

**ICD - 10 Based IQ Score (Clinicians' Assessment)**	
Mean	30.71 ± 11.00

### Distribution of the children in different diagnostic categories of ICD - 10 criteria for mental retardation [17] based on care-givers and clinicians' judgments

Four (9.1%), 27 (61.4%), 11 (25.0%) and 2 (4.5%) of the children with intellectual disability were classified under the ICD - 10 categories of F 70, F 71, F 72, and F 73 respectively as assessed by both the clinicians' IQ scores and calculated 'ratio IQ' scores.

Table 3 showed the distribution of the children under different ICD - 10 diagnostic categories [17] based on clinicians' IQ scores assessment and calculated 'ratio IQ' scores. There was no significant difference in the distribution of IQ diagnostic categories among the children based on clinicians' assessment and 'ratio IQ' scores calculation (χ^2 ^= 0.00, df = 3, p = 1.000).

**Table 3:** Distribution of the children with intellectual disability in different diagnostic categories of mental retardation based on ICD - 10 criteria

**Category**	**Mental Retardation**	**IQ Range**	**Median IQ Scores**	**Mean 'Ratio IQ' Scores**	**Classification Based on Clinicians' Assessment** **N (%)**	**Classification Based on 'Ratio IQ' Scores** **N (%)**
**F 70**	Mild	50 - 70	60	55.0 ± 7.1	4 (9.1)	4 (9.1)

**F 71**	Moderate	35 - 49	42	44.1 ± 7.3	27 (61.4)	27 (61.4)

**F 72**	Severe	20 - 34	27	36.1 ± 12.8	11 (25.0)	11 (25.0)

**F 73**	Profound	0 - 20	10	27.3 ± 15.3	2 (4.5)	2 (4.5)

Correlations between mean IQ scores as assessed by the clinicians, using the median IQ scores in each diagnostic category and mean 'ratio IQ' scores as calculated from ratio of estimated mental age and chronological age of each child.

### Zero order correlation

When the chronological/actual ages of the children were not controlled for, the correlation between clinicians' assessed IQ scores and 'ratio IQ' scores was statistically significant (r = 0.47, df = 42, p = 0.001). The zero order correlation co-efficient is shown in Table 4.

**Table 4:** Zero order correlation between clinicians' assessed IQ scores and 'ratio IQ' scores

**Variables**	**'Ratio IQ' Scores**	**Clinicians Assessed IQ Scores**	**Chronological/Actual Ages of the Children**
**'Ratio IQ' Scores**	r = 1.00, df = 0	r = 0.47, df = 42, p = 0.001	r = - 0.31, df = 42, p = 0.039

**Clinicians Assessed IQ Scores**	r = 0.47, df = 42, p = 0.001	r = 1.00, df = 0	r = 0.49, df = 42, p = 0.001

**Chronological/Actual Ages of the Children**	r = - 0.31, df = 42, p = 0.039	r = 0.49, df = 42, p = 0.001	r = 1.00, df = 0

### First order correlation

When the chronological/actual ages of the children are controlled for, the correlation between clinicians' assessed IQ scores and 'ratio IQ' scores showed a much higher level of statistical significance (r = 0.75, df = 41, p = 0.000).

### Histograms and normal curve distributions of IQ scores as assessed by the clinicians and the 'ratio IQ' scores calculated from ratio of estimated mental age and chronological age of the children

**Figure 1:**
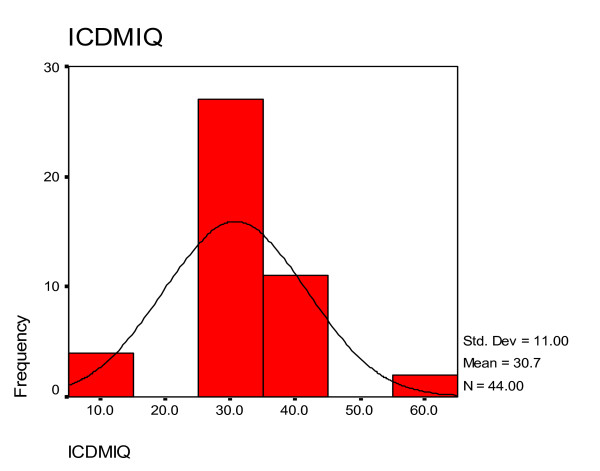
**Histogram and normal curve distribution of IQ scores as assessed by the clinicians based on ICD - 10 criteria for mental retardation**. ICDMIQ - IQ based on ICD - 10 criteria for mental retardation.

**Figure 2:**
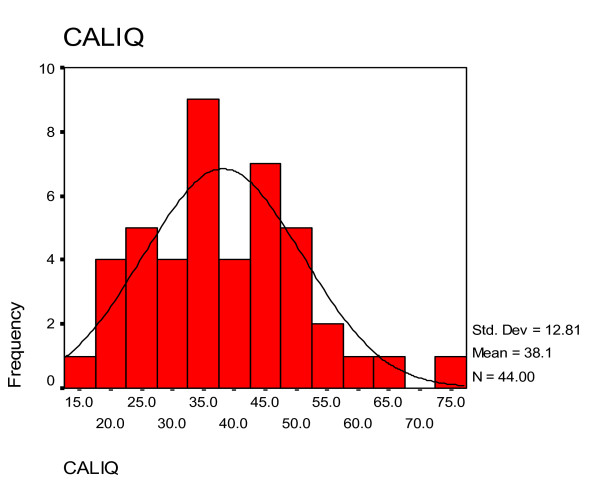
**Histogram and normal curve distribution of 'ratio IQ' scores as calculated from the ratio of estimated mental age and chronological age of the children**. CALIQ - IQ calculated based on ratio of estimated mental age to chronological age.

Figure 1 showed the histogram and normal curve distribution of IQ scores among the children using median scores in each diagnostic category for mental retardation specified in ICD - 10 diagnostic criteria based on clinicians' assessment. Figure 2 showed the histogram and normal curve distribution of 'ratio IQ' scores among the children.

## Discussion

Both the clinicians' assessment of IQ scores and 'ratio IQ' scores calculation based on mental age as estimated by the care-givers showed agreement in classification of the children into different diagnostic categories of mental retardation as specified by ICD - 10 [17]. The correlations between mean IQ score as assessed by the clinicians and mean 'ratio IQ' score were significant with and without controlling for the chronological age of the children.

The observation that cultural background could influence neural activity that underlies both high and low level cognitive function [14] indicate the need for culture appropriate assessment of human intelligence, especially in sub-Saharan Africa where great multicultural diversity is the case [15,16].

Special educators providing educational instructions to children with intellectual disability may need a quick assessment of a child IQ score in sub-Saharan African sub-cultures, where there is wide variation in cultural practices and societal structures. Employing a ratio of culture-specific estimated mental age and chronological age of the child would provide a viable option in the absence of culture appropriate standardized 'deviance IQ' tests like Wechsler Intelligence Scales for Children (WISC - R, WISC - III and WISC - IV) used widely among children in the western parts of the world.

## Limitations

Both the clinicians' assessment of IQ scores and estimation of mental age of the children by the care-givers from which the 'ratio IQ' scores were derived were subjective. However, the effect of this subjectivity had been cushioned by allowing two independent assessors with the same socio-cultural background with the children in each case of the clinicians and care-givers to reach a consensus on a particular child following the criteria highlighted in the procedure section. The 'ratio IQ' scores produced for each child from the estimation of mental age would serve an approximate guide in educational needs assessment of these children and for purpose of research.

Another limitation of mental age estimation in children as proposed by this study is that, lack of background knowledge of normal child development by the care-givers may constitute an impediment in estimation of mental age. It is therefore recommended that people in position of caregivers willing to estimate mental age of children with intellectual disability according to the procedure highlighted in this study have some background knowledge of normal child development.

## Conclusion

In view of the confounding cultural and environmental influence on human intelligence [9-14], there would be need to put into consideration cultural background when assessing intelligence scores in children coming from various cultures.

Therefore, for the purpose of educational needs assessment and research in sub-Saharan African children with intellectual disability, consensus estimation of mental age in relative to peculiar socio-cultural milieu of the child would provide a viable option of estimating IQ score through the ratio of estimated mental age and chronological age of the child, in the absence of culture appropriate standardized 'deviance IQ' tests.

## Competing interests

The authors declare that they have no competing interests.

## Authors' contributions

All authors contributed to the conception of the study. MOB was involved in writing the initial draft of the manuscript. MOB, VNU, INO, CMA and POE were involved in revising the manuscript. All authors read and approved the final draft of the manuscript.
